# Antibiotic-Resistant *Desulfovibrio* Produces H_2_S from Supplements for Animal Farming

**DOI:** 10.3390/microorganisms11040838

**Published:** 2023-03-25

**Authors:** Olga V. Karnachuk, Alexey V. Beletsky, Andrey L. Rakitin, Olga P. Ikkert, Marat R. Avakyan, Viacheslav S. Zyusman, Andrei Napilov, Andrey V. Mardanov, Nikolai V. Ravin

**Affiliations:** 1 Laboratory of Biochemistry and Molecular Biology, Tomsk State University, 634050 Tomsk, Russia; 2 Institute of Bioengineering, Research Center of Biotechnology of the Russian Academy of Sciences, Leninsky Prosp, Bld. 33-2, 119071 Moscow, Russia

**Keywords:** antibiotic resistance, resistance plasmids, sulfate-reduction, swine manure, gut microbiota, *Desulfovibrio*

## Abstract

Sulphate-reducing bacteria, primarily *Desulfovibrio*, are responsible for the active generation of H_2_S in swine production waste. The model species for sulphate reduction studies, *Desulfovibrio vulgaris* strain L2, was previously isolated from swine manure characterized by high rates of dissimilatory sulphate reduction. The source of electron acceptors in low-sulphate swine waste for the high rate of H_2_S formation remains uncertain. Here, we demonstrate the ability of the L2 strain to use common animal farming supplements including L-lysine-sulphate, gypsum and gypsum plasterboards as electron acceptors for H_2_S production. Genome sequencing of strain L2 revealed the presence of two megaplasmids and predicted resistance to various antimicrobials and mercury, which was confirmed in physiological experiments. Most of antibiotic resistance genes (ARG) are carried by two class 1 integrons located on the chromosome and on the plasmid pDsulf-L2-2. These ARGs, predicted to confer resistance to beta-lactams, aminoglycosides, lincosamides, sulphonamides, chloramphenicol and tetracycline, were probably laterally acquired from various *Gammaproteobacteria* and *Firmicutes*. Resistance to mercury is likely enabled by two *mer* operons also located on the chromosome and on pDsulf-L2-2 and acquired via horizontal gene transfer. The second megaplasmid, pDsulf-L2-1, encoded nitrogenase, catalase and type III secretion system suggesting close contact of the strain with intestinal cells in the swine gut. The location of ARGs on mobile elements allows us to consider *D. vulgaris* strain L2 as a possible vector transferring antimicrobials resistance determinants between the gut microbiote and microbial communities in environmental biotopes.

## 1. Introduction

Studies of antibiotic resistance genes (ARG) have mainly focused on pathogenic and commensal bacteria and paid less attention to environmentally relevant microorganisms. Sulphate-reducing bacteria (SRB) are a functional guild of prokaryotes of significant environmental importance due to the end product of their metabolism, hydrogen sulfide. Anaerobic SRB are common inhabitants of natural and technogenic ecosystems, such as marine and freshwater sediments, groundwater, soils and wastewater treatment facilities. SRB, primarily members of the genus *Desulfovibrio*, also occur in the animal and human intestine [1,2,3,4,5,6,7,8]. Possible associations of SRB with pathologies, including ulcerative colitis [3,9,10], inflammatory bowel disease [11], colorectal cancer [12,13], bacteremia [4,14,15,16,17], brain abscess [18] and renal infection [19], are discussed in the scientific literature. A recent study revealed a pathogenic effect of *Desulfovibrio* in the gut on fatty liver in diet-induces obese mice and children with obesity [20]. Little is known about resistance to antibiotics in sulphate-reducing bacteria and the reported cases refer to clinical practice. *D. desulfuricans* isolated from an immunocompetent patient with bacteremia was susceptible to penicillin (MIC of 4 μg/mL) and other antimicrobial agents [4]. The 36 clinical isolates of *Desulfovibrio* evaluated at the Mayo Clinic from 1997 to 2013, which included *D. legallii*, *D. desufuricans*, *D. fairfieldensis*, *D. intestinalis* and *D. piger*, were susceptible to penicillin, metronidazole and carbapenems [21]. *Desulfovibrio* spp. were highly susceptible to sulbactam-ampicillin, meropenem, clindamycin, metronidazole and chloramphenicol, but generally showed high MICs to piperacillin and piperacillin-tazobactam [22].

Plasmids often contribute to the spread of ARG via horizontal gene transfer between unrelated bacteria [23]. The occurrence of plasmids is not often reported in *Desulfovibrio*. The classical studies of J.R. Postgate reported the presence of megaplasmids in *D. gigas*, *D. desulfuricans* and *D. vulgaris* [24]. Genome sequences of megaplasmids from *D. vulgaris* Hildenborough revealed plasmid-encoded nitrogen fixation, a type-III secretion system, and catalase [25]. The *D. vulgaris* plasmid encoding nif genes may be lost when the organism is cultivated in ammonium-containing media. The plasmid lacks homologs to previously characterized plasmid replication or partitioning genes. A small plasmid (8568 bp), pNC1, has been reported in *D. africanus* subsp. *uniflagellum* [26]. The cloning vector was constructed on the base of pNC1 [27]. Furthermore, 53.6% of the plasmid contains genes associated with replication, mobilization, and partitioning and its compatible hosts include *D. africanus* and *Pseudomonas aeruginosa* PA14. 

SRB in wastewater treatment facilities can contribute to the undesirable continuous H_2_S formation [28]. Our previous study demonstrates that a low abundance SRB community in manure slurry from a large-scale swine finishing facility produces up to 7.25 nmol reduced S cm^−3^ day^−1^ and plays an important role in malodorous H_2_S production [29]. A microcosm experiment revealed that the sulphate reduction in manure was limited by the sulfate concentration. Suggested sources of sulphate, an electron acceptor for its dissimilatory reduction, may include animal feed supplements, as well as solid animal bedding often including gypsum and plasterboard. The CaSO_4_ use as a solid-phase electron acceptor by *Desulfovibrio* results in production of H_2_S concentrations that are compatible with those produced from soluble NaSO_4_ [30]. Lysine is the first-limiting amino acid in swine diets, and most of its supplements are formulated as L-lysine hydrochloride or L-lysine sulphate [31]. The sulphate from lysine supplements, as well sulphate moiety of gypsum or plasterboard can provide a significant amount of substrate for the dissimilatory SO_4_ reduction to H_2_S. 

Hydrogen sulphide production in swine manure slurry has been linked to *Desulfovibrio* spp. [29]. The *Desulfovibrio* in swine manure may originate from the pig gut. Recent reports have confirmed that *Desulfovibrio* is the dominant SRB in pig intestine [32,33]. A new species, *Desulfovibrio porci,* has been isolated from pig feces [34]. Despite being banned in the EU and some other countries, antibiotics, including tetracycline, streptomycin and sulfonamides, are still used in animal husbandry to promote the growth of livestock [35] and, consequently, lead to the selection of a resistant gut microbiome. In our previous study, two *Desulfovibirio*, *D. desulfuricans* L4 and *D. vulgaris* L2, were isolated from swine manure slurry [29]. Analysis of the *Desulfovibrio* L4 genome revealed the presence of 10,876 bp long plasmid designated pDsulf-L4, containing a multidrug-resistance cassette consisting of the tetracycline resistance gene *tetA* (MFS family exporter), streptomycin resistance genes *strA-strB* (aminoglycoside phosphotransferases) and sulfonamide-resistance dihydropteroate synthase gene *sul2*. A horizontal acquisition of pDsulf-L4 from *Shigella flexneri* harboring the identical plasmid [36] has been suggested. The plasmid horizontal transfer could occur in the gut and was followed by the spread of drug-resistant *D. desulfuricans* L4 in the environment. 

The presence of antibiotic resistance genes in *D. vulgaris* L2, as well as its ability to produce H_2_S from animal farming supplements such as L-lisyne suphate, gypsum and gypsum-containing plasterboard, remain unresolved. This study aims to characterize the physiology of the L2 strain with a focus on SO_4_-containing animal farming supplements and elucidate the antibiotic resistance mechanisms through genome sequencing.

## 2. Materials and Methods

### 2.1. Physiological Tests 

The L2 strain was isolated from the settlement lagoon at a large swine finishing facility, as described previously [29]. Briefly, cumulative manure slurry from the settlement lagoon was used as the inoculum for pure culture isolation. Liquid Widdel–Bak (WB) medium with formate as an electron donor supplemented with zero-valent iron was chosen to prevent overgrowth of other heterotrophic microorganisms. Cell morphology was observed by phase contrast microscopy using an Axio Imager A1 microscope and by transmission electron microscopy (TEM) of ultra-thin sections prepared as previously described [37]. 

Physiological tests were carried out in the modified WB medium that contained (per litre) 4.0 g Na_2_SO_4_, 0.2 g KH_2_PO_4_, 0.25 g NH_4_Cl, 1 g NaCl, 0.4 g MgCl_2_·6H_2_O, 0.5 g KCl, 0.113 g CaCl_2_, 2 mL of vitamin solution, 1 mL of trace element solution, 1 mL each of Na_2_SeO_3_ (final concentration of 23.6 µM) and Na_2_WO_4_ (24.2 µM) solutions. Vitamin and trace element solutions were prepared as described by *Widdel* and *Bak* [38]. Each cultivation vial received an iron wire (100% Fe) as described previously [39,40]. Lactate (18 mM) was used as an electron donor. Growth was tested at incubation temperatures in the range of 4–50 °C. Growth was determined by microscopic cell counts in triplicate samples. Specific growth rates were calculated from the cell counts during the exponential phase of growth. Further physiological experiments were conducted at 37 °C.

Growth was analyzed with the following electron donors: 7.5 mM formate, 9 mM acetate, 13.5 mM propionate, 7 mM butyrate, 7 mM pyruvate, 4.5 mM succinate, 9 mM fumarate, 7.5 mM malate, 5 mM citrate, 1 mM palmitate, 5 mM fructose, 5 mM glucose, 3 mM sucrose, 25 mM ethanol, 17 mM propanol, 13.5 mM butanol, 11 mM glycerol, and 1 g L^−1^ peptone and tryptone (all Sigma-Aldrich, Saint Louis, MO, USA). Carbohydrate stock solutions were sterilized using polyethersulfone 0.22 µm Millex-GP filter units (Merck Millipore, Darmstadt, Germany). If growth was observed, the culture was subcultured at least five times in the presence of each electron donor and acceptor to confirm their utilization. 

**Figure 1:**
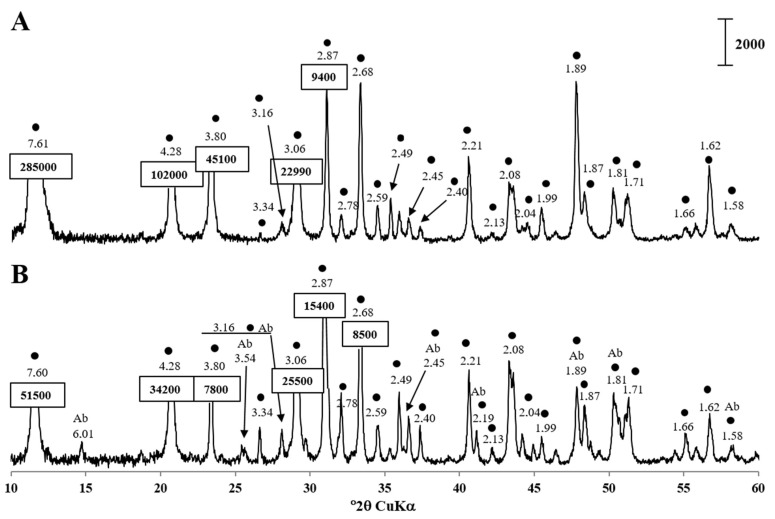
X-ray diffraction patterns of (**A**) gypsum and (**B**) gypsum plasterboard. The diagnostic peaks for gypsum CaSO_4_ 2H_2_O (●) are indicated. Letter code: Ab = albite, NaAlSi_3_O_8_. The vertical bar shows the scale of relative counts.

The tested soluble electron acceptors were the following: 28 mM sodium sulfate, 2 mM sodium sulfite, 20 mM sodium sulfite, 20 mM sodium thiosulfate, 10 mM fumarate and 5 mM calcium nitrate. Solid-phase electron acceptors, gypsum and gypsum plasterboard, were sterilized as powder by autoclaving. Each 12 mL culture vial received 100 mg of gypsum or gypsum plaster board. X-ray diffraction did not reveal impurities in the used gypsum specimen (Figure 1A). Plasterboard contained gypsum as the dominant phase, an alumosilacte, albite (Figure 1B). All the acceptors were tested in the presence of 18 mM lactate.

### 2.2. Sequencing of Desulfovibrio vulgaris L2 Genome

Genomic DNA was extracted from strain L2 cells using a Power Soil DNA Isolation Kit (MO BIO Laboratories, Carlsbad, CA, USA). The library for Illumina sequencing was prepared using the TruSeq Nano DNA Library Prep Kit (New England Biolabs, Ipswich, MA, USA). Sequencing on the Illumina MiSeq generated 4,590,696 paired-end reads (2× 300 nt, ~2.2 Gbp in total). Overlapping paired-end reads were merged using FLASH v.1.2.11 [41], and low-quality bases were trimmed using Sickle v.1.33 (https://github.com/najoshi/sickle; accessed on 28 January 2023).

Genomic DNA of strain L2 was additionally sequenced on the MinION (Oxford Nanopore, Oxford, UK) using a Ligation Sequencing Kit 1D protocol and an R9.4 flow cell (FLO-MIN106). Sequencing resulted in 74,516 reads with a total length of ~552 Mbp. *De novo* assembly of MinION reads was performed using Flye v.2.7b [42], Illumina reads were mapped to the assembly and used for the correction with three iterations of Pilon v.1.22 software [43]. Three circular contigs of 3,611,640 bp, 175,461 bp and 109,048 bp, representing a chromosome and two plasmids were obtained. Gene search and annotation were performed using the RAST server 2.0 [44].

## 3. Results

### 3.1. Desulfovibrio vulgaris L2 Physiology and Resistance to Antibiotics

**Figure 2:**
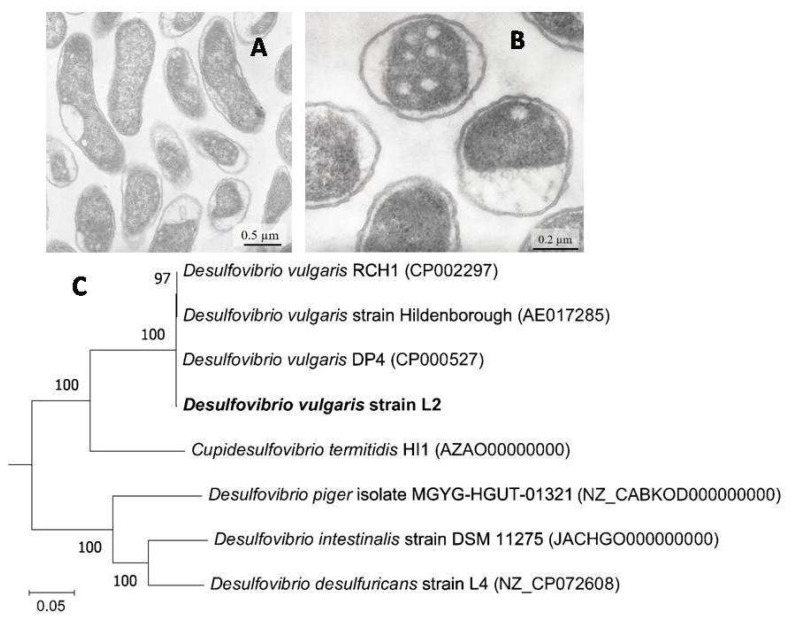
(**A**,**B**) TEM micrographs of ultrathin layers of strain L2 and (**C**) Phylogenetic position of *D. vulgaris* strain L2 inferred using neighbor-joining inference on the 120 concatenated single copy marker proteins. The optimal tree is shown. The percentage of replicate trees in which the associated taxa clustered together in the bootstrap test (1000 replicates) are shown next to the branches. The tree is drawn to scale, with branch lengths in the same units as those of the evolutionary distances used to infer the phylogenetic tree. The evolutionary distances were computed using the Poisson correction method and are in the units of the number of amino acid substitutions per site. All ambiguous positions were removed for each sequence pair (pairwise deletion option). There was a total of 5040 positions in the final dataset. Evolutionary analyses were conducted in MEGA11.

Cells of strain L2 are slightly curved rods, 1.6–3.0 μm long, and 0.4–0.6 μm wide (Figure 2A,B). The 16S rRNA gene sequence of strain L2 was 99.78% similar to that of *Desulfovibrio vulgaris* Hildenorough (recently reclassified as *Nitratidesulfovibrio vulgaris* [45]). The sequence similarity was above of 98.7%, the species boundary cutoff, assuming that L2 is a novel strain of *D. vulgaris*. The 16S rRNA sequences of strain L2 was 100% similar to that of *Desulfovibrio vulgaris* DP4, isolated from heavy metal-impacted lake sediment [46]. The phylogenetic position of strain L2 was confirmed by its genome analysis. The average nucleotide identity (ANI) between L2 genome and *D. vulgaris* Hildenorough was 99.06%, a value above the species boundary cutoff of 95% [47,48]. Strain L2 was capable of growth at temperatures between +15 and +45 °C with an optimum temperature of 37 °C. Strain L2 used lactate, pyruvate, fumarate, succinate, ethanol, glycerol, peptone and tryptone as electron donors for sulphate reduction. Limited growth was observed with malate, fructose and formate. The strain did not grow with citrate, butyrate, propionate, benzoate, palmitate, glucose, sucrose, butanol and propanol. 

**Figure 3:**
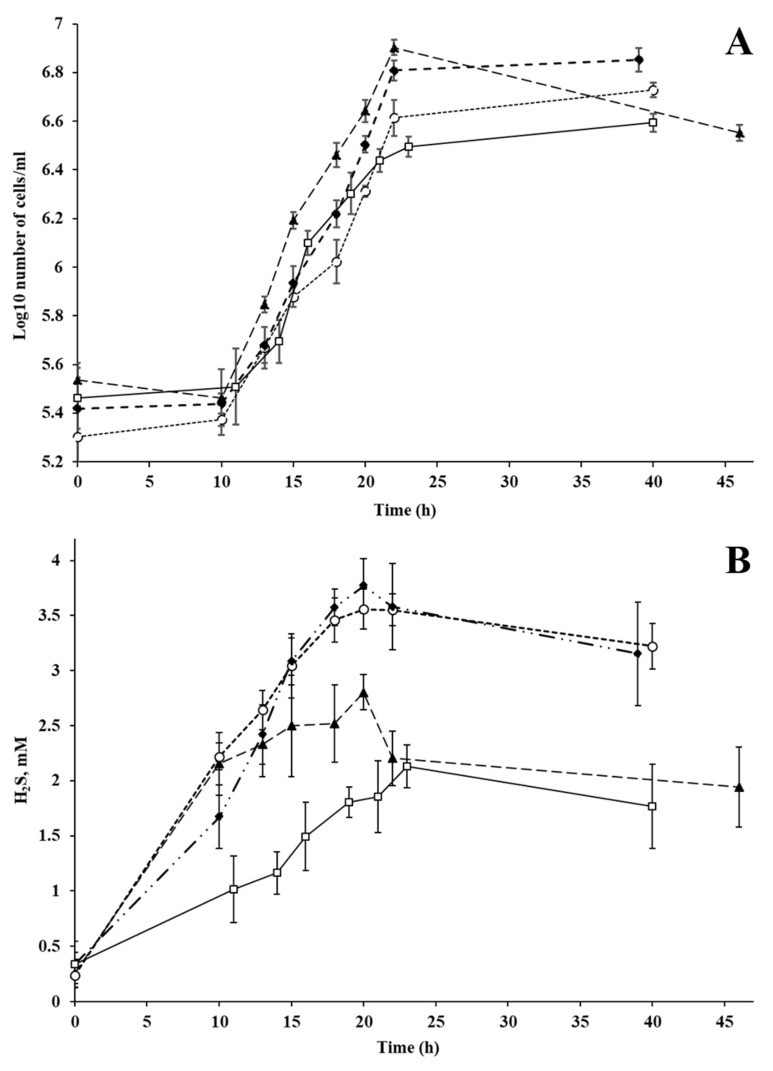
(**A**) Growth of strain L2 with Na_2_SO_4_, gypsum (CaSO_4_ 2H_2_O), gypsum plasterboard and L-lysine sulphate (2(C_6_H_14_N_2_O_2_). H_2_SO_4_) as the sole electron acceptor and (**B**) H_2_S production during growth with Na_2_SO_4_, gypsum (CaSO_4_. 2H_2_O), gypsum plasterboard and L-lysine sulphate (2(C_6_H_14_N_2_O_2_). H_2_SO_4_): ▲ Na_2_SO_4_ (triangles); ♦ gypsum (diamonds); ○ gypsum plasterboard (circles); □ L-lysine sulphate (squares).

Sulfite, thiosulfate, nitrate and fumarate, in addition to sulphate, could be used as electron acceptors. Strain L2 could grow with gypsum, gypsum plasterboard, and L-lysine sulphate as the sole electron acceptor with the specific growth rates of 0.15–0.18 h^−1^ (Figure 3A). Sulphide formation from gypsum and plasterboard reached 3.77 ± 0.24 and 3.56 ± 0.18 mM, respectively (Figure 3B). The maximum sulphide concentration formed from L-lysine sulphate was 2.12 ± 0.19.

Strain L2 was resistant to streptomycin, tetracycline, ampicillin, kanamycin and gentamicin. Minimum inhibitory concentrations (MIC) were as follows (in micrograms per milliliters): streptomycin, 800; tetracycline, 450; ampicillin, 750; kanamycin, 350; and gentamicin, 100. The strain could grow at Hg^+^ concentrations not exceeding 7 μg/mL. 

### 3.2. General Genome Properties 

The genome of strain L2 consists of a chromosome of 3,611,640 bp and two circular plasmids 175,461 and 109,048 nucleotides long, designated pDsulf-L2-1 and pDsulf-L2-2, respectively. The chromosome contains all the genes required for dissimilatory sulfate reduction in *Desulfobacterota*, namely, sulfate permease, sulfate adenylyl transferase (*sat*), adenylsulfatereductase (*aprAB*) and dissimilatory sulfitereductase (*dsrABD* and *dsrC*). Also present are the genes for the adenylsulfatereductase-associated electron transport complex, QmoABC, heterodisulfidereductase, and the sulfite reductase-associated electron transport proteins, DsrMKJOP. The presence of several molybdopterin-dependent oxidoreductases of the Psr family is consistent with the ability of strain L2 to use alternative electron acceptors for anaerobic respiration. Moreover, the genome of strain L2 also contains cytochrome c oxidase, which can be involved not only in oxygen detoxification, but also in respiration under microaerophilic conditions.

The larger plasmid, pDsulf-L2-1, was highly similar (>97% nucleotide sequence identity over >95% length) to plasmids found in *D. vulgaris* strains RCH1 (plasmid pDEVAL01), Hildenborough (plasmid pDV) and DP4 (plasmid pDVUL01). These strains are closely related to L2 (Figure 2C) and belong to the same species. pDsulf-L2-1 contains Type IC CRISPR/Cas system with 32 spacer-repeat units and all the essential genes of a type III secretion system. Type III secretion systems are commonly used by pathogenic bacteria to inject effector proteins directly into the host cell cytoplasm to influence host responses [49]. Since the L2 strain presumably originates from the gut of animals, this plasmid may be involved in the interaction of these bacteria with intestinal epithelial cells. Considering metabolically important functions, the plasmid contains the MoFe nitrogenase genes, the *katE* catalase gene and the *chrBA* chromate resistance operon.

Plasmids similar to pDsulf-L2-2 have not been found in any of the *Desulfovibrio* species. Although sequences highly similar to some regions of pDsulf-L2-2 (about 10% of the total length) were found in various gamma-proteobacteria, a GeneBank search did not reveal sequences similar to the entire pDsulf-L2-2. The plasmid encodes several site-specific recombinases, transposases and toxin-antitoxin systems, as well as proteins involved in conjugative transfer and mobilization.

### 3.3. Genetic Determinants of Antibiotic Resistance 

**Figure 4:**
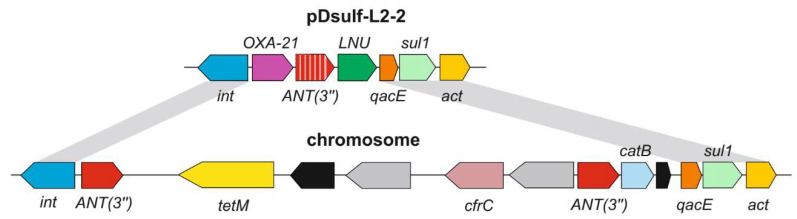
Integrons carrying antibiotic resistance genes on the chromosome and plasmid pDsulf-L2-2. Genes are represented by arrows. Transposase genes are shown by gray arrows; black arrows indicate genes encoding the conjugate transfer protein and the hypothetical protein. Gray areas highlight regions of sequence identity (>99%) between the plasmid and the chromosome.

A notable feature of the pDsulf-L2-2 plasmid is the presence of two gene clusters that can confer antibiotic resistance. The first is a class 1 integron [50] containing six resistance genes (Figure 4). A search in the CARD database predicted that they encode the beta-lactamase of the OXA-2 family conferring resistance to beta-lactams such as carbapenem, cephalosporin and penams, the aminoglycoside nucleotidyltransferase of the ANT(3″) family responsible for the inactivation of aminoglycosides, the lincosamidenucleotidyltransferase (LNU), the QacE efflux pump of the MFS superfamily conferring resistance to antiseptics, sulfonamide-resistant dihydropteroate synthase Sul1, and acetyltransferase(*act*) distantly related to SAT-2 streptothricinacetyltransferases and aminoglycoside acetyltransferase of the AAC(6′) family. Close homologues of these genes, including 100% identical integronintegrases, have been found in gamma-proteobacteria (*Escherichia, Salmonella, Pseudomonas*, etc.). The second closely located locus contains genes for the regulatory protein TetR and the tetracycline efflux pump Tet(C).

The chromosome of strain L2 also contains a class 1 integron with an integrase completely identical to that of the pDsulf-L2-2 integron (Figure 4). However, the genes content of this integron is different, and its genes seem to be obtained from different sources. The first gene encodes an aminoglycoside nucleotidyltransferase of the ANT(3″) family; it differs from the ANT(3″) gene located on the plasmid, but a GeneBank search revealed identical genes in some *Enterobacteriaceae*. The integron is then interrupted by an insert containing the oppositely oriented genes encoding the tetracycline-resistant ribosomal protective protein Tet(M), a conjugal transfer protein, two transposases, and the 23S rRNA methyltransferase CfrC conferring resistance to linezolid and phenicol antibiotics. Genes identical to *tet(M)* and *cfrC* were found in *Firmicutes*, *Streptococcus pyogenes* HKU419, and *Clostridioides difficile* 020696, respectively. The next is the second copy of the first gene of this integron, ANT(3″), it is followed by *catB* chloramphenicol acetyltransferase gene. This region is identical to the p035_A-VIM-1 plasmid of *Klebsiella aerogenes* 035. The next three genes are identical to the last three genes of the pDsulf-L2-2 plasmidintegron, *qacE, sul1*, and *act*.

Another chromosomal locus contains genes for the regulatory protein TetR and the tetracycline efflux pump Tet(C). The sequences of these genes differed from the corresponding genes of the plasmid pDsulf-L2-2. However, a nearly identical region was found on the pDsulf-L4 plasmid of *D. desulfuricans* strain L4 isolated from the same manure storage lagoon.

### 3.4. Genetic Determinants of Resistance to Mercury

Plasmid pDsulf-L2-2 contains a mercuric resistance operon *merRTPADE* containing the genes for the regulatory protein MerR, the transport protein MerT, the periplasmic Hg^2+^—binding protein MerP, the mercuric ion reductase MerA, the transcription regulator MerD, the transport protein MerE and the protein containing the EAL domain. Such *mer* operons allow bacteria to detoxify Hg^2+^ by enzymatic reduction into volatile metallic mercury [51]. In plasmid pDsulf-L2-2 the *mer* operon is probably part of a transposon, since it is followed by genes for the resolvase and the Tn3 family transposase. Sequenced genomes of other *Desulfovibrio* species lacked similar *mer* operons, but gene clusters with identical nucleotide sequences are present in the chromosomes and plasmids of several species of *Pseudomonas* and *Aeromonas*.

Interestingly, the second mer operon is located on the chromosome of the L2 strain close to the tetR-tetC locus. This operon has a similar gene order and content (*merRTPCADE)*, with the exception of an additional merC gene for the mercury transport protein located between merP and merA. The nucleotide sequence identity between the plasmid and chromosomal mer operons is only 81–85%, indicating their independent acquisition by lateral transfer rather than transfer between pDsulf-L2-2 and the chromosome. Identical regions spanning both mer and tetR-tetC were found in plasmids and chromosomes of various *Enterobacteriaceae* (*Salmonella enterica*, *Escherichia coli*, *Klebsiella pneumonia*, *Klebsiella enterica*, etc.), indicating a single acquisition event.

## 4. Discussion

The results of our study suggest that *D. vulgaris* L2 isolated from swine manure originates from the intestinal microbial community. Recent metagenome studies provide evidence that *Desulfovibrio* is the dominating group of sulphate reducers in the swine intestine. *Desulfovibrio* were revealed to be the most abundant SRB in piglet cecum by using *DsrA*-targeted analysis [32]. At the species level, *D. intestinalis* was the predominant SRB in Meishan and Yorkshire piglets. *Desulfovibrio piger* was the second abundant SRB in Meishan piglets. The authors hypothesized that the *Desulfovibrio* role in the gut was intestinal hydrogen removal. A study of the pig intestinal microbiome using fecal samples sequencing and composite genome (MAG) assembly showed that the genes encoding dissimilatory sulfite reductase were only identified in MAGs belonging to the *Desulfovibrionaceae* [33]. SRB have long been recognized as environmentally relevant prokaryotes and major players in carbon and sulphur cycles [28]. There is a growing amount of evidence that SRB, primarily *Desulfovibrio*, is an important constituent of microbial community in the animal intestine [1,2,3,4,5,6,7,8]. 

The involvement of *Desulfovibrio* in various pathologies has been documented, but the mechanisms remain largely unknown [8,17]. Nie with co-authors [52] showed that *D. fairfieldensis* damages epithelial barriers and activate inflammation and pyroptosis in macrophages via outer membrane vesicles. The occurrence of type III secretion system (T3SS) on the pDsulf-L2-1 plasmid, revealed in our study, suggests its possible close contact with intestinal epithelial cells. T3SS is used by a number of pathogens, such as *Escherichia coli*, *Salmonella*, *Yersinia* and *Pseudomonas* [53], as well as commensal bacteria, including *Rhizobium* [54], to deliver effector proteins into eukaryotic host cells. A ncbi blastp search reveals a number of ATPase SctN closely related to that found in pDsulf-L2-1 in other *Desulfovibrio* species. Catalase, which plays a role in oxidative stress, has been identified as a putative T3SS effector shared between plant and animal pathogens [55]. The catalase occurrence on pDsulf-L2-1 close to T3SS corresponds to a possible *D. vulgaris* contact with epithelial cells. 

Until now, the resistance determinants to tetracycline and other antimicrobials applied in animal husbandry have not been detected in SRB. Several reports of *Desulfovibrio* susceptibility to clinically relevant antibiotics are based on physiological tests [4,21,22] and do not decipher the occurrence of antibiotic resistance determinants in species genomes. 

The observed resistance of strain L2 to a number of antibiotics is most likely determined by laterally acquired resistance genes identified on the chromosome and plasmid pDsulf-L2-2. Aminoglycoside nucleotidyltransferase of the ANT(3″) family and aminoglycoside acetyltransferase of the AAC(6′) family are probably responsible for resistance to streptomycin, kanamycin and gentamicin. The observed resistance to ampicillin is probably due to beta-lactamases of the OXA-2 family. All of these resistance genes are located on a class 1 integron. Such integrons play a key role in the spread of antibiotic resistance because they can capture and express various resistance genes and are often located on plasmids and transposons facilitating their lateral transfer [50,56]. The observed resistance to tetracycline is likely determined by the efflux pump Tet(C) and the ribosomal protection protein Tet(M).

All of these resistance determinants were acquired by horizontal gene transfer from various *Gamma-proteobacteria* and *Firmicutes*, as evidenced by the presence of identical genes in these lineages and the absence of their close homologues in other *Desulfovibrio* species. The only exception is a region of about 2.4 kb containing *tetR-tetC*, which is common to the chromosome of strain L2 and the plasmid pDsulf-L4 of *D. desulfuricans* strain L4 isolated from the same site. This short (10876 bp) plasmid additionally contains *strA-strB* (aminoglycoside phosphotransferase) streptomycin resistance genes and the *sul2* dihydropteroate synthase sulfonamide resistance gene [29], which are absent in strain L2. Since the *tetR-tetC* locus in the L2 strain chromosome is linked to the *mer* operon, and the entire cluster was obtained from a member of the *Enterobacteriaceae* family, its independent acquisition by the L4 strain from the same donor is the most plausible scenario, although transfer from the L2 strain chromosome to the plasmid pDsulf-L4 of strain L4 cannot be ruled out. 

Strain L2 belongs to *D. vulgaris*, a model SRB. The *D. vulgaris* Hildenborough strain was isolated by J. R. Postgate in 1946 from clay near Hildenborough, Kent (UK) [24]. Two other strains with available genomes originate from arsenic contaminated soil (DP4) [46] and chromium bioremediation site in Hanford, WA [57]. All these strains contain plasmids similar to pDsulf-L2-1, but only the L2 strain harbors an additional resistance plasmid.

H_2_S is considered as one of the most important pollutants associated with livestock production [58]. The high sulphate reduction rates by *Desulfovibrio* measured with a radioactive tracer in manure slurry at a large swine finishing facility [29] left open the question of the source of the electron acceptor for H_2_S production. Our experiments with *D. vulgaris* L2 have shown that the gypsum and gypsum plasterboard used for animal bedding constitute a large reservoir of solid-phase sulphate that is used by *Desulfovibrio* and other SRB for their metabolism. The L2 strain grows at the same rate and produces the same amount of H_2_S from gypsum or gypsum plasterboard as it does from soluble sulphate. 

Currently, most of the supplemental lysine used in pig diets is in the form of hydrochloride, which contains 78.8% of lysine; more recently, lysine sulphate containing ≥54.6% of lysine has been introduced as an alternative source of supplemental lysine [31]. Copper sulfate (CuSO_4_) has been used as a supplement to reduce the incidence of diarrhea and improve growth performance of piglets over the past decades [59]. L-lysine sulphate and other food supplements formulated as sulphate salts can increase the H_2_S production in the animal gut.

## Data Availability

The GenBank accession number for the 16S rRNA gene sequences of *Desulfovibrio vulgaris* strain L2 is OP445610. The annotated genome sequences of *Desulfovibrio vulgaris* strain L2 have been deposited in the GenBank database under the accession numbers CP116246 (chromosome), CP116248 (plasmid pDsulf-L2-1) and CP116247 (plasmid pDsulf-L2-2), BioProject PRJNA924109.
